# Solvent-Induced Morphology Control of Polymer Assemblies with Improved Photothermal Features

**DOI:** 10.1021/jacs.5c08355

**Published:** 2025-07-22

**Authors:** Yingtong Luo, Jianhong Wang, Yudong Li, Işıl Yeşil Gür, Marco M.R.M. Hendrix, Yiğitcan Sümbelli, Alexander Fusi, Ilja K. Voets, Loai K. E. A. Abdelmohsen, Jingxin Shao, Jan C. M. van Hest

**Affiliations:** † Bio-Organic Chemistry, Institute for Complex Molecular Systems (ICMS), 3169 Eindhoven University of Technology, P.O. Box 513, 5600MB Eindhoven, The Netherlands; ‡ Self-Organizing Soft Matter, Department of Chemical Engineering and Chemistry & Institute of Complex Molecular Systems, Eindhoven University of Technology, P.O. Box 513, 5600MB Eindhoven, The Netherlands

## Abstract

Organic photothermal agents (OPTAs) are extensively utilized in applications such as therapy and imaging. However, enhancing their photothermal performance often depends on complex molecular designs, which are limited by the challenges of chemical synthesis. Herein, we present a straightforward strategy to optimize the optical absorbance of OPTAs by adjusting the morphology of assemblies of an amphiphilic block copolymer (PEG_44_-PTA_5_), leading to enhanced photothermal conversion efficiency. By changing the polarity of the organic solvent in which the polymer was dissolved, addition of water to induce assembly led to the exclusive formation of polymersomes or bicontinuous nanospheres. The morphological variations were confirmed using a range of electron microscopy techniques and small-angle X-ray scattering. Due to their mesoporous structure, the bicontinuous nanospheres exhibited superior light-harvesting capabilities, achieving a high absorption coefficient of 1.1 × 10^5^ M^–1^ cm^–1^ and a photothermal conversion efficiency of 45% when irradiated with a 808 nm laser. Our work introduces a facile solvent-induced assembly strategy for precisely controlling the morphology of OPTAs while simultaneously tuning their light absorption properties to enhance photothermal conversion.

## Graphical abstract

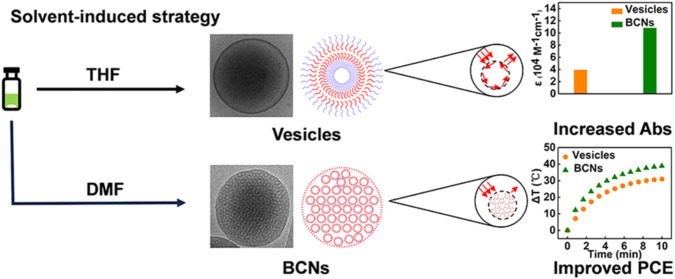

## Introduction

Organic photothermal agents (OPTAs) have attracted increasing attention over the past few decades due to their good biocompatibility, versatile chemical modification, and efficient photothermal conversion.^1–6^ These features make them highly suitable for a wide range of applications, including photothermal therapy, photoacoustic imaging, and water purification.^7–10^ Therefore, developing OPTAs with high photothermal conversion efficiency (PCE) is of significant importance. In principle, heat generation primarily depends on effective light absorption and nonradiative energy dissipation. To enhance PCE, molecular engineering is commonly employed to optimize these properties. For example, Shao and co-workers expanded the π-conjugated structure of donor–acceptor–donor (D–A–D) OPTAs, enhancing intramolecular charge transfer (ICT) and facilitating a bathochromic-shift in the absorption spectra, leading to more effective photothermal conversion.^11^ Similarly, Liu et al. introduced molecular rotors and bulky alkyl chains into the central D–A core of OPTAs to promote nonradiative decay.^12^ Although these molecular design strategies offer valuable approaches for enhancing PCE, they often require intricate design and complex molecular synthesis, significantly limiting their practical applications. Therefore, developing facile strategies to enhance PCE is of great interest for advancing OPTAs.

The photothermal properties of plasmonic metal materials (e.g., Au, Ag, Pt, Pd, and Cu) are strongly influenced by their size, shape, and morphology.^13–15^ By adjusting these factors, inorganic nanomaterials can exhibit diverse physicochemical properties. For example, modifying their morphology into rod-like, cage-like, star-like, or flower-like structures enhances their photothermal conversion through the localized surface plasmon resonance (LSPR) effect.^16,17^ However, such morphology-dependent strategies for improving PCE have rarely been explored in OPTAs. Recently, photothermal responsive polymersomes (PTA-polymersomes) composed of amphiphilic block copolymers have been developed as OPTAs for anticancer treatment.^18^ Their practical applicability remained limited due to insufficient light-harvesting capacity. In this regard, bicontinuous nanospheres (BCNs) assembled from amphiphilic block copolymers, which feature a complex internal network arranged in a regular lattice, offer a promising alternative.^19,20^ Their mesoporous structure and high specific surface area have made them widely utilized in the photocatalysis field.^21,22^ These structures enhance light utilization efficiency due to multiple light reflections within their inner channels.^23^

Notably, the phase space for bicontinuous structures is very narrow in block copolymer phase diagrams.^24^ It is therefore essential to carefully design the polymers to have the appropriate ratio of hydrophilic to hydrophobic segments. This makes the construction of these BCNs complex. A critical parameter for morphological control is the hydrophilic block ratio (*f*), defined as the hydrodynamic volume of the hydrophilic block relative to the total hydrodynamic volume of the copolymer. Notably, *f* is influenced not only by the block length ratio but also by the choice of organic solvent used to dissolve the copolymer prior to water addition. By adjusting the solvent, *f* can be fine-tuned postsynthetically, enabling access to the narrow phase space required for bicontinuous morphology, even for copolymers with slightly off-target compositions.^25^ The composition of the organic solvent significantly influences polymer dimensions during self-assembly, thereby affecting the equilibrium morphology.^26^ McKenzie et al. for example, provided valuable insights into the role of solvent selection in their study of the self-assembly of poly­(ethylene oxide)-*b*-poly­(octadecyl methacrylate) (PEO-*b*-PBMA), demonstrating that BCNs can form by adjusting the relative block proportions and using a nonselective cosolvent.^27^ Common solvents such as N,N-dimethylformamide (DMF), tetrahydrofuran (THF), and dioxane have distinct dielectric constants (ε) of 36.7, 7.6, and 2.3, respectively, serving as indicators of solvent polarity and their interaction strength with polymer chains. This variation in polarity allows precise control over solvent–polymer interactions during self-assembly, enabling fine-tuning of morphology by adjusting the solvent’s affinity for the polymer chains.^28–30^

In this work, we investigated if we could improve the PCE of polymer-based OPTAs by controlling their morphology via solvent engineering. For this purpose, the assembly behavior of two block copolymers, PEG_44_-PTA_3_ and PEG_44_-PTA_5_ with varying hydrophobic segment lengths was systematically studied as a function of the polarity of the organic solvent. PEG_44_-PTA_5_ was able to form diverse uniform morphologies, including vesicles, fused vesicles and BCNs (Scheme 1). Notably, compared to vesicles, BCNs exhibited a significantly enhanced photothermal performance due to their superior light harvesting capability. This study therefore presents a facile strategy to enhance the photothermal performance of OPTAs by morphological control.

**Scheme 1:**
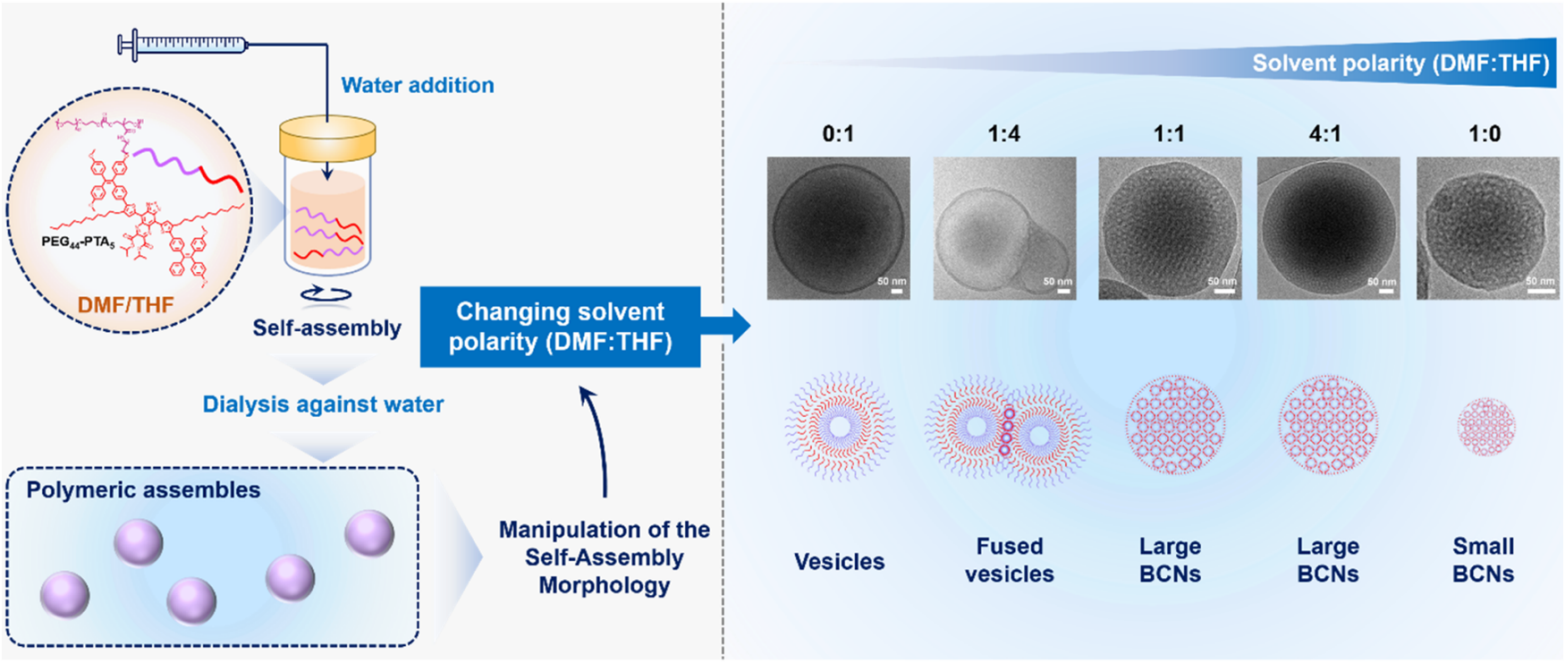
Schematic Illustration of Morphological Control by Solvent Polarity Adjustment during Self-Assembly^a^

a TEM images and corresponding schematic cartoons depict the morphological transformations as solvent polarity increases.

## Results and Discussion

In previous work, we synthesized a photothermal block copolymer (PEG_44_-PTA_2_) that successfully self-assembled into photothermal responsive polymer vesicles (PTA-polymersomes).^18^ The hydrophilic block ratio (*f*) is a key parameter influencing the morphology after assembly.^31–33^ To achieve bicontinuous structures, the copolymer must exhibit significant block asymmetry, with a substantially larger hydrophobic block. In many block copolymer systems, the optimal *f* value typically falls below 0.25, though this range varies depending on the polymer’s chemical composition and architecture.^34–37^ With a relatively high *f* value of 0.53, PEG_44_–PTA_2_ assembled in THF was unsuitable for forming bicontinuous nanostructures (BCNs).^19^ To address this issue, we increased the hydrophobic component and synthesized PEG_44_-PTA_3_ (*f* = 0.35) and PEG_44_-PTA_5_ (*f* = 0.21). Detailed polymer synthesis and characterization, including nuclear magnetic resonance spectroscopy and size exclusion chromatography, are provided in the Supporting Information (Scheme S1, Figures S1–S10 and Table S1).

The organic solvent that dissolves both the hydrophobic and hydrophilic blocks also plays a crucial role in determining the morphology of the colloids. Eisenberg et al. demonstrated that various morphologies could be formed in an amphiphilic block copolymer system by varying the solvent composition, including *N*,*N*-dimethylformamide (DMF), tetrahydrofuran (THF), dioxane and their mixtures.^29,38–40^ We subsequently used the block copolymers PEG_44_-PTA_3_ and PEG_44_-PTA_5_ to create diverse polymeric nanoarchitectures by adjusting the organic solvent composition. Initially, polymer solutions with varying organic solvent ratios were prepared at a concentration of 2 mg/mL. Water was then incrementally added via a syringe pump at a rate of 0.25 mL/h until it reached 50 vol %. The self-assembled particles were obtained by dialyzing the mixtures against a large volume of water. The resulting colloidal morphologies were analyzed using small-angle X-ray scattering (SAXS), scanning electron microscopy (SEM), transmission electron microscopy (TEM), and cryo transmission electron microscopy (Cryo-TEM). Systematic variation of the dioxane-to-THF ratio in the initial solution led to distinct differences in the assembled structures. Notably, a 1:1 cosolvent ratio yielded besides individual vesicles several interconnected structures by bicontinuous networks (Figure 1a). When the ratio was increased to 1:4, the higher THF content led to an increase in overall solvent polarity. This elevated initial polarity enhanced repulsion between the hydrophilic chains, thereby destabilizing the membrane.

**Figure 1:**
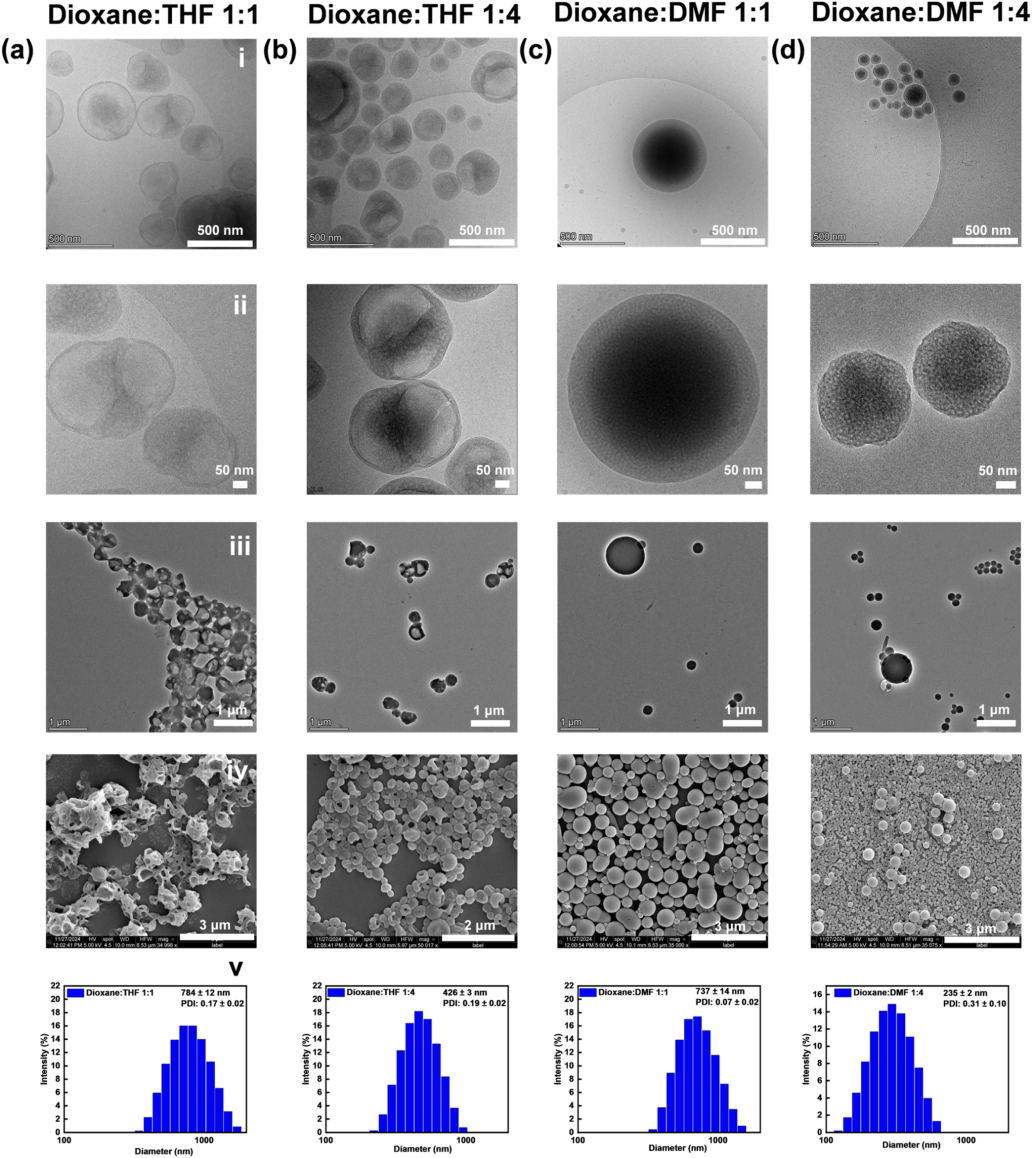
Morphological characterization of self-assembled PEG_44_-PTA_5_ structures prepared using different cosolvent ratios (v/v%) by cryo-TEM (i), zoomed-in cryo-TEM (ii), TEM (iii), SEM (iv) and size distribution histogram measured by DLS (v). (a) dioxane/THF = 1:1, (b) dioxane/THF = 1:4, (c) dioxane/DMF = 1:1, (d) dioxane/DMF = 1:4.

Consequently, unilamellar vesicles nearly disappeared, and vesicles with a bicontinuous structure were mainly observed (Figure 1b). This transformation is driven by the system’s tendency to minimize surface free energy, promoting the formation of a complex bicontinuous phase or vesicle fusion.^41–43^ To examine whether predominantly BCNs with uniform size could be achieved, we replaced THF with DMF, a solvent with a higher dielectric constant (ε), as the cosolvent. Using a dioxane/DMF ratio of 1:1, large BCNs (737 ± 14 nm) were obtained, whereas a ratio of 1:4 resulted in smaller BCNs (235 ± 2 nm), though with a broader size distribution (PDI = 0.31 ± 0.1) (Figures 1c,d, S11 and Table S2). Although the use of a dioxane/DMF solvent system promoted BCN formation, closer inspection revealed poor dispersity and partial aggregation/fusion of the structures, leading to inhomogeneous morphologies (Figure S12). These observations highlight that neither condition yields ideal uniform BCNs, prompting further optimization in the following section. To enhance the colloidal dispersity and morphological uniformity of BCNs, we further increased the solvent polarity by using THF and DMF as cosolvents. To evaluate the resulting nanostructures, cryo-TEM was employed to capture the native morphology of the particles in aqueous solution, while SEM and TEM were used to assess their morphology under dry conditions, providing complementary structural information. Similar to the results obtained with dioxane/THF, pure THF also led to the formation of two distinct vesicle types: individual vesicles and fused vesicles with a bicontinuous structure (Figure 2a). As the DMF content in THF increased to 25 vol %, nearly all vesicles became interconnected, forming a bicontinuous structure (Figure 2b). This is further supported by DLS data, which showed a 1.5-fold increase in average particle size compared to self-assemblies formed in pure THF (Table S2). With increasing solvent polarity, we observed the formation of large bicontinuous nanospheres with an average diameter of 550 ± 5 nm (Figure 2c). Further increasing the DMF content slightly reduced the average diameter to 535 ± 1 nm without altering the morphology (Figure 2d). Ultimately, small bicontinuous nanospheres with an average diameter of 263 ± 4 nm were assembled in pure DMF (Figure 2e). Furthermore, the three types of nanoparticles remained stable with no significant size changes observed over 10 days, suggesting their suitability for long-term storage (Figure S13). Internal domain analysis revealed that the internal pore sizes of the large and small PTA-BCNs were 9 ± 1 nm and 8 ± 2 nm, respectively. The zeta potential of PTA-polymersomes, large and small PTA-BCNs was determined to be −24.6 ± 0.3, −38.9 ± 0.5, and −12.3 ± 0.7 mV, respectively (Figure S14). Although the copolymers do not contain any ionizable groups, these negative surface potentials likely arise from the adsorption of hydroxide ions at the particle–water interface. This phenomenon is commonly observed in neutral block copolymer systems and can be attributed to the presence of polar aromatic and heteroaromatic units (e.g., benzothiadiazole and thiophene) in the PTA block, which increases interfacial polarity and facilitates ion adsorption.^44,45^

**Figure 2:**
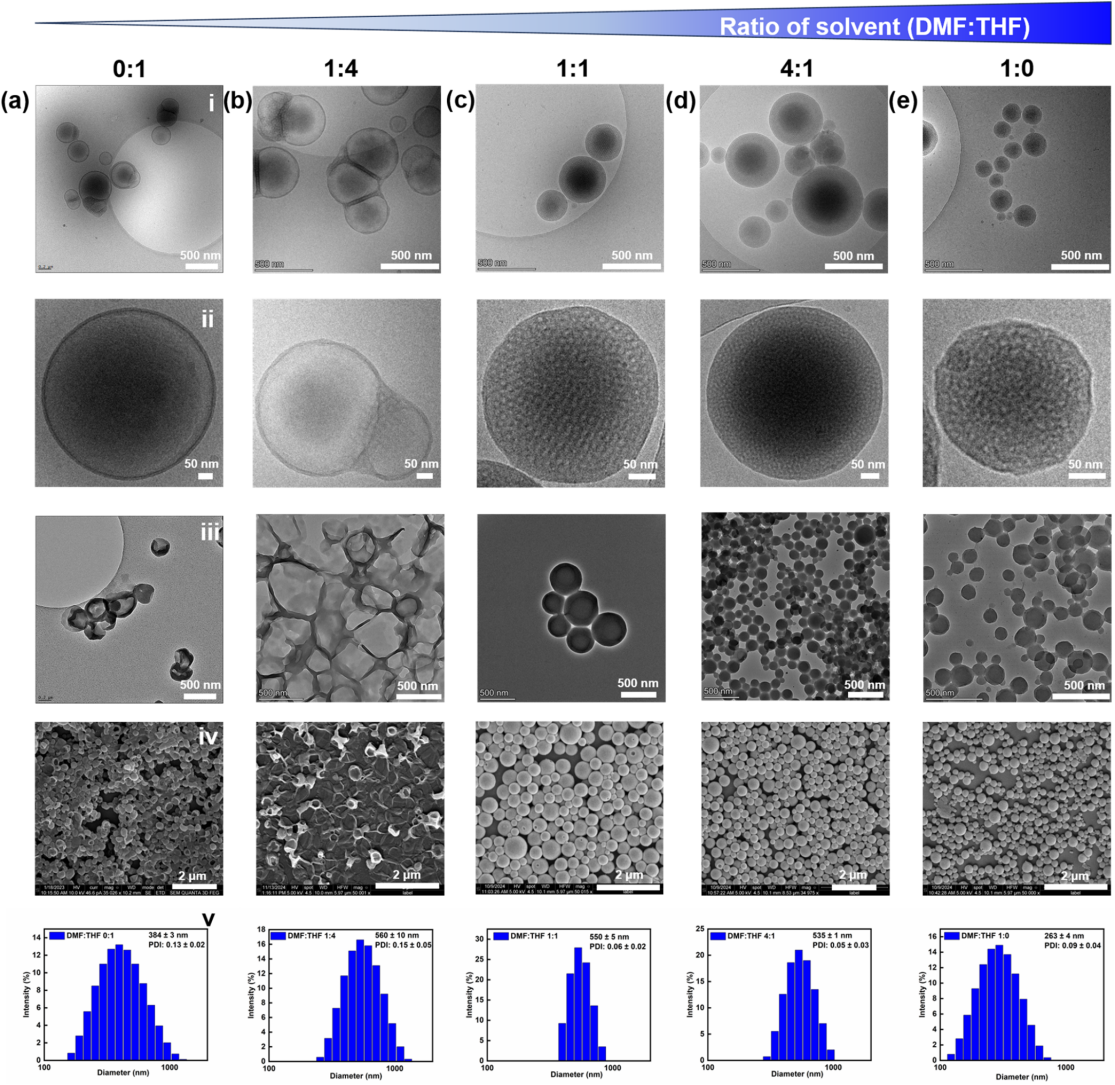
Morphological characterization of self-assembled PEG_44_-PTA_5_ structures prepared using different DMF/THF cosolvent ratios (v/v%) by cryo-TEM (i), zoomed-in cryo-TEM (ii), TEM (iii), SEM (iv) and size distribution histogram measured by DLS (v). (a) 0:1 (b) 1:4 (c) 1:1 (d) 4:1 (e) 1:0.

To investigate the potential correlation between polymer composition and morphology, PEG_44_-PTA_3_, which has a relatively high *f* value, was studied in a DMF/THF cosolvent system (Figure S15). When the DMF content was below 50 vol %, vesicles were predominantly observed in the assemblies (Figure S15a,b). As the ratio increased to 1:1, both vesicles and BCNs coexisted in the solution, differing from the assemblies of PEG_44_-PTA_5_ under the same conditions (Figures 2c, S15c and S16). The delayed formation of BCNs can be attributed to the higher *f* value of PEG_44_-PTA_3_ compared to PEG_44_-PTA_5_, which reduces the tendency for phase separation and structural reorganization. With a further increase in DMF content to 75%, BCNs and micelles coexisted after dialysis (Figure S15d). Notably, when pure DMF was used as the initial solvent, PEG_44_-PTA_3_ tended to form tube-like assemblies with a bicontinuous structure. Although BCNs could be assembled from PEG_44_-PTA_3_, their size and morphology remained irregular (Figures S15e, S17 and Table S3).

In conclusion, a morphological transformation in the self-assembled structure of PEG_44_-PTA*_n_* was observed, transitioning from vesicles to BCNs as the affinity between the cosolvent and polymer increased. This transformation was achieved by adjusting the DMF content in the cosolvent and modifying the hydrophobic block length of the copolymer (Figure S18). Therefore, both solvent polarity and polymer composition jointly influence the morphology and size of the assemblies. The interactions between polymer chains and the solvent are critical factors influencing the morphology of assemblies. On one hand, the interaction between the hydrophobic block and the solvent determines the degree of PTA chain stretching. An increase in solvent polarity reduces the stretching of the PTA block, causing the PTA chains to collapse. While the collapse of the hydrophobic chains reduces their hydrodynamic volume, the resulting chain compaction may enhance local intermolecular interactions-such as π-π stacking or hydrophobic association which in turn facilitates the formation of a bicontinuous phase within the membrane.^46–48^ On the other hand, enhanced repulsive interactions between hydrophilic chains increase membrane tension, ultimately promoting vesicle fusion.^49–52^ In summary, these two factors collectively facilitate the formation of BCNs.

In polymersomes, light is primarily reflected and refracted within the internal aqueous core, resulting in a short optical path, which limits their ability to effectively capture light energy. In contrast, BCNs exhibit superior light-capturing capabilities due to their unique bicontinuous structure. Composed of two interconnected phases (hydrophilic and hydrophobic), this structure forms a multichannel system that enhances light scattering and reflection within the particle. As a result, the optical path is extended, allowing light to interact more with the material which improves light absorption efficiency.^53,54^ Together, these factors make BCNs significantly more effective in capturing and converting light energy compared to vesicles (Figure S19).^23,55^ The internal structures of vesicles and BCNs were confirmed by cryo-TEM. The electron contrast of the internal structures was determined through grayscale analysis. Typical bilayer structures were observed in vesicles, while bicontinuous structures were seen in BCNs (Figure 3a–d). SAXS analysis further confirmed the internal organization of the self-assemblies. No distinct peaks were observed for PTA-polymersomes, which is consistent with the typical vesicular structure (Figure 3e). In contrast, Bragg reflections for the large PTA-BCNs displayed relative spacing ratios of 1:√3:2:√7 (Figure 3f), suggesting the presence of a hexagonal structure (*p*6*mm*) phase.^36,56–58^ The scattering profiles of the small PTA-BCNs (Figure 3g) showed similarly positioned Bragg peaks, although with significantly weaker intensity, suggesting the presence of the same hexagonal structure but with lower internal ordering. This result agrees with cryo-TEM analysis showing predominant formation of a bicontinuous internal network in BCNs, compared with a bilayer structure in vesicles (Figure S20). All PTA-based polymeric nanoparticles (PTA-polymersomes, large and small PTA-BCNs) showed remarkable absorption in the near-infrared (NIR) region due to the presence of the PTA moieties (Figure 3h). To further confirm that BCNs enhance light adsorption through their porous structure, we calculated the absorption coefficient at 808 nm for the different assemblies. The results showed that both large and small PTA-BCNs had higher absorption coefficients (ε) than PTA-polymersomes, with the small PTA-BCNs displaying a particularly high ε of 1.1 × 10^5^ M^–1^ cm^–1^ at 808 nm, 2.8 times higher than that of PTA-polymersomes (Figure S21). Moreover, the extremely low fluorescence intensity of the nanoparticles suggests good photothermal properties upon 808 nm laser irradiation (Figure S22).

**Figure 3:**
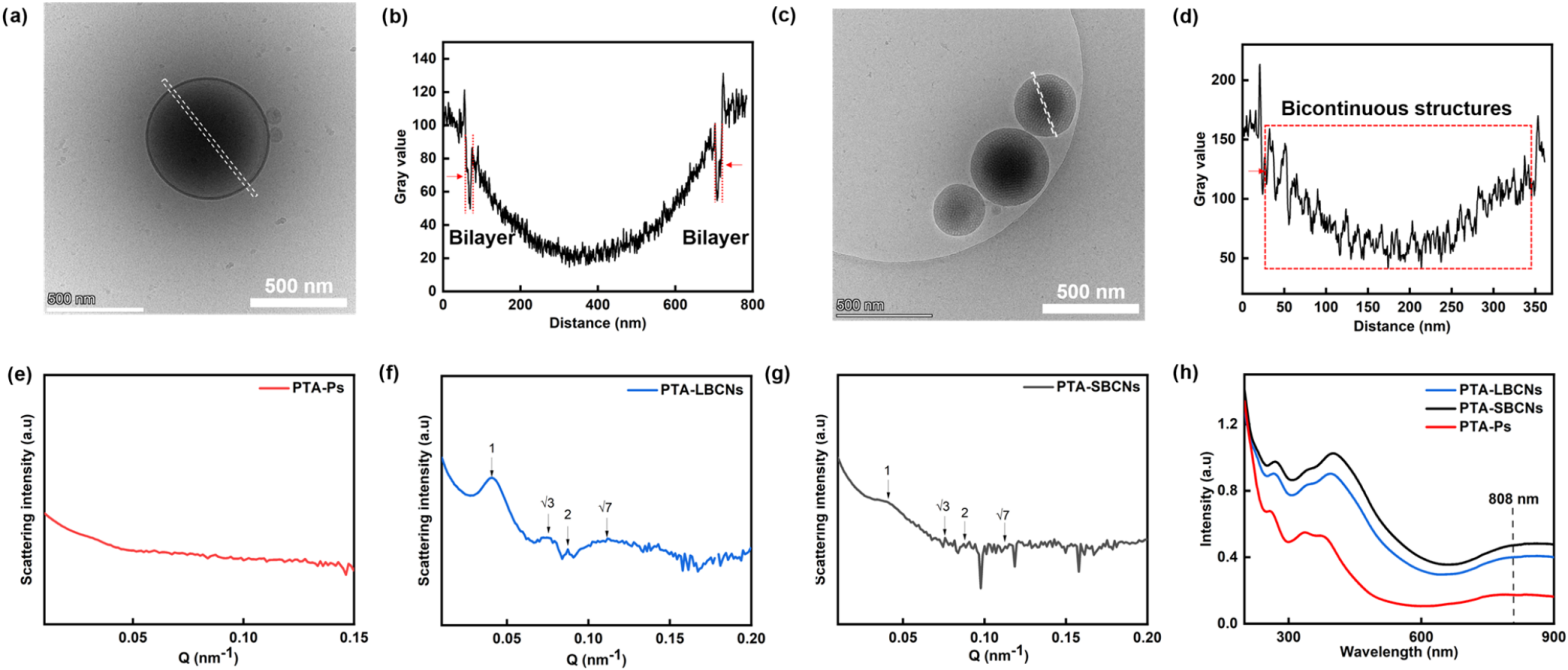
(a) Cryo-TEM image of vesicles with the white dashed line representing the measured greyscale. (b) Plot of the gray values measured in (a). (c) Cryo-TEM image of BCNs with the white dashed line representing the measured greyscale. (d) Plot of the gray values measured in (c). (e–g) SAXS scattering of PTA-polymersomes (PTA-Ps), large PTA-BCNs (PTA-LBCNs) and small PTA-BCNs (PTA-sBCNs), respectively (20 mg/mL). (h) Absorption spectra of PTA-polymersomes, large PTA-BCNs and small PTA-BCNs in water (0.05 mg/mL). The dashed line indicates the position of 808 nm, which corresponds to the NIR laser wavelength used for photothermal evaluation.

We found that the absorption properties of the fabricated nanoparticles are closely dependent on their morphology, and consequently, their photothermal effect is also morphology-dependent. First, the temperature change upon NIR laser irradiation (808 nm) of the three different nanoparticles (PTA-polymersomes, large PTA-BCNs, and small PTA-BCNs) was investigated (Figure 4a). The maximum temperature increase observed for PTA-polymersomes, similar to our previously synthesized photothermal responsive polymersomes, was 31.0 °C. In contrast, large and small PTA-BCNs raised the temperature by 36.0 and 39.0 °C, respectively, both higher than PTA-polymersomes. The photothermal conversion efficiency (PCE) of the small PTA-BCNs (45.0%) and large PTA-BCNs (43.8%) was higher than that of the PTA-polymersomes (35.2%) (Figures S23–S25). Furthermore, the PCE of the small PTA-BCNs was found to be dependent on both the output laser intensity and nanoparticle concentration (Figure 4b,c), indicating that the photothermal effect can be easily regulated. Notably, at each concentration, PTA-BCNs induced a higher temperature increase than PTA-Ps, further highlighting the benefit of their enhanced PCE in potential biomedical applications (Figure 4c). Next, we evaluated the photothermal stability of the small PTA-BCNs via cyclic heating–cooling measurements. As shown in Figure 4d, the temperature increase remained stable after 5 cycles, demonstrating good photothermal stability. The photothermal heating of each group upon laser irradiation was also visually observed with an IR camera, as shown in Figure 4e. Furthermore, the structural stability of the small PTA-BCNs after 10 min of laser irradiation (1 W) was evaluated. As shown in Figures S26 and S27, the morphology and size of the nanoparticles remained intact after the treatment. Consequently, we can conclude that the small PTA-BCNs exhibit good thermal adjustability and stability under laser irradiation. Additionally, to the best of our knowledge, this is the first report of photothermal responsive BCNs.

**Figure 4:**
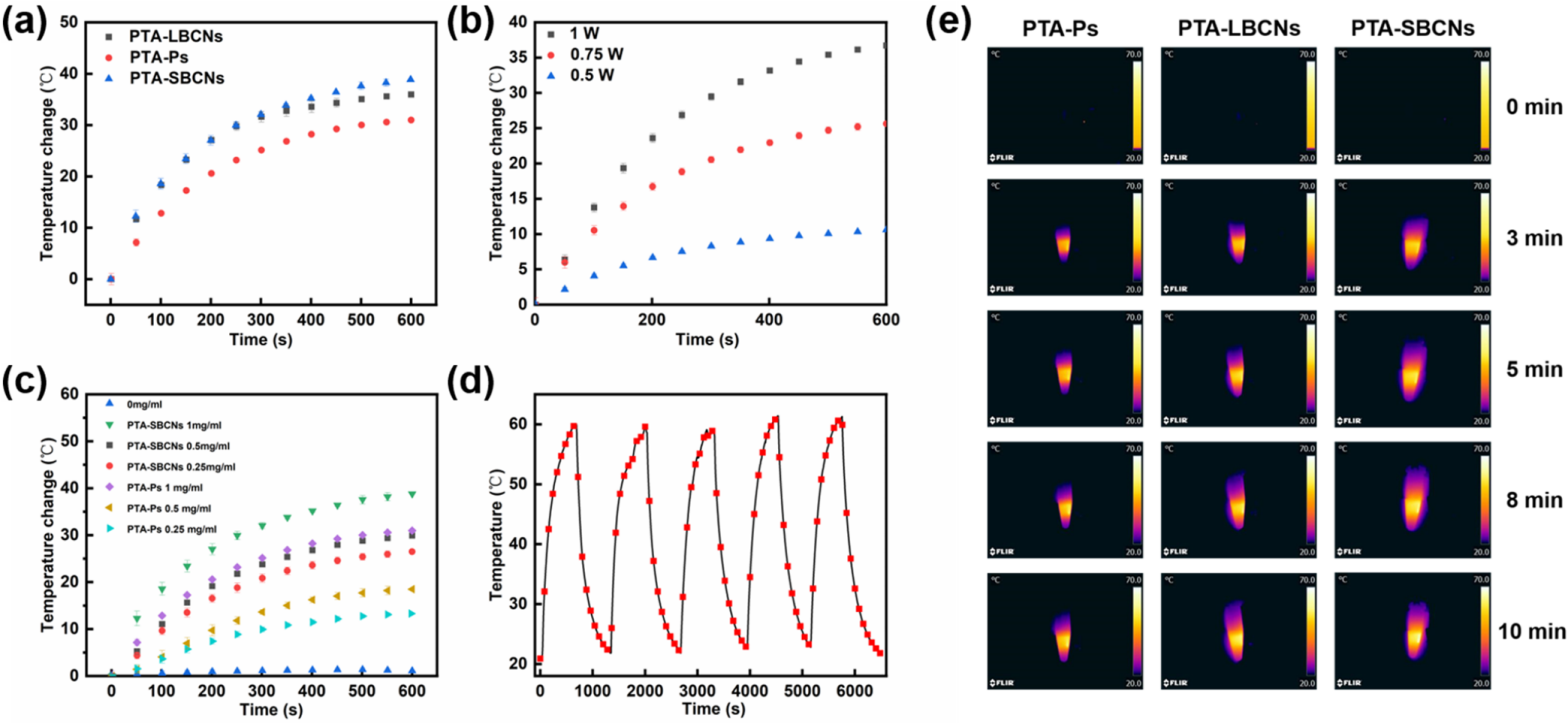
Photothermal properties of PTA-polymersomes, small PTA-BCNs and large PTA-BCNs. (a) Temperature change of PTA-polymersomes, small and large PTA-BCNs in water (1 mg/mL) upon NIR laser irradiation (808 nm, 1 W) for 10 min. (b) Temperature change of small PTA-BCNs in water (1 mg/mL) under 808 nm laser irradiation for 10 min at different power levels (0.5, 0.75, and 1 W). (c) Temperature change of small PTA-BCNs and PTA-Ps in water at varying concentrations (0, 0.25, 0.5, and 1 mg/mL) under 808 nm laser irradiation (1 W) for 10 min. (d) Photothermal stability of small PTA-BCNs in water (1 mg/mL) during five circles of heating–cooling. (e) Corresponding infrared thermal mappings of PTA-polymersomes, small and large PTA-BCNs in water (1 mg/mL) upon 808 nm laser irradiation (1 W) as a function of time.

## Conclusions

In summary, we have demonstrated that morphological control during the assembly of photothermally responsive block copolymers leads to improved photothermal conversion efficiency (PCE). This control is achieved by adjusting the composition of the solvent in which the polymers are initially dissolved, as interactions between the polymer chains and the solvent play a key role in governing morphological transitions. In particular photoresponsive bicontinuous nanospheres enhance light capture, significantly improving PCE. Unlike traditional molecular engineering approaches, this morphology-manipulation strategy offers a simpler alternative, eliminating the need for complex molecular design and synthesis. Overall, we expect that this solvent-induced strategy will provide a facile and effective route to photothermally responsive organic nanoparticles with improved PCE.

## Supplementary Material



## Notes

Detailed experimental conditions and methods; molecular synthesis details; self-assemblies preparation; PCE calculation; SAXS analysis; ^1^H NMR spectra; ^19^F NMR spectra; GPC analysis; emission spectra; DLS analysis; SEM images; TEM images; photothermal curves (PDF)
