# Supplementary material for: Solvent-Induced Morphology Control of Polymer Assemblies with Improved Photothermal Features
Source: J Am Chem Soc. 2025 Jul 22;147(31):28189–97. doi: 10.1021/jacs.5c08355 (PMC12333373; doi:10.1021/jacs.5c08355)
Supplement: Supplementary file 1 [file ja5c08355_si_001.pdf]

Supporting Information

Solvent-Induced Morphology Control of Polymer Assemblies with Improved Photothermal Features

Yingtong Luo,[1] Jianhong Wang,[1] Yudong Li,[1] Işıl Yeşil Gür,[1] Marco M.R.M. Hendrix,[2] Yiğitcan Sümbelli,[1] Alexander Fusi,[1] Ilja K. Voets,[2] Loai K. E. A. Abdelmohsen*,[1] Jingxin Shao*,[1] and Jan C. M. van Hest*,[1]

[1]Bio-Organic Chemistry, Institute for Complex Molecular Systems (ICMS), Eindhoven University of Technology, P.O. Box 513, 5600MB Eindhoven, The Netherlands

[2]Self-Organizing Soft Matter, Department of Chemical Engineering and Chemistry & Institute of Complex Molecular Systems, Eindhoven University of Technology, P.O. Box 513, 5600 MB, Eindhoven, The Netherlands

*Corresponding should be address to: L. K. E. A. Abdelmohsen@tue.nl; J.Shao@tue.nl; J.C.M.v.Hest@tue.nl

Contents

1. Materials

2. Instruments

3. Methods

3.1 Synthesis of photothermal block copolymer (PEG_{44}-PTA_3 and PEG_{44}-PTA_5).

3.2 Preparation of self-assemblies *via* solvent switch method.

3.3 Photothermal performance of PTA-Ps, PTA-LBCNs and PTA-SBCNs.

3.4 Calculation of photothermal conversion efficiency.

4. Supplementary schemes, figures and table

Figure S1. Representative ^{1}H NMR spectrum of TMCP (400 MHz, 298 K, $CDCl_3$).

Figure S2. Representative ^{19}F NMR spectrum of TMCP (376 MHz, 298 K, $CDCl_3$).

Figure S3. Representative ^{1}H NMR spectrum of PEG_{44}-$TMCP_3$ (400 MHz, 298 K, $CDCl_3$).

Figure S4. Representative ^{19}F NMR spectrum of PEG_{44}-$TMCP_3$ (376 MHz, 298 K, $CDCl_3$).

Figure S5. Representative ^{1}H NMR spectrum of PEG_{44}-$TMCP_5$ (400 MHz, 298 K, $CDCl_3$).

Figure S6. Representative ^{19}F NMR spectrum of PEG_{44}-$TMCP_5$ (376 MHz, 298 K, $CDCl_3$).

Figure S7. Representative ^{19}F NMR spectrum of PEG_{44}-$PTMCP_5$ (top), PEG_{44}-PTA_5 before purification (middle) and PEG_{44}-PTA_5 after purification (down).

Figure S8. ^{1}H-NMR spectrum of PEG_{44}-PTA_3 (400 MHz, 298 K, $CDCl_3$).

Figure S9. ^{1}H-NMR spectrum of PEG_{44}-PTA_5 (400 MHz, 298 K, $CDCl_3$).

Figure S10. GPC trace of PEG_{44}-$PTMCP_3$, PEG_{44}-$PTMCP_5$, PEG_{44}-PTA_3 and PEG_{44}-PTA_5.

Table S1. GPC analysis of block copolymers.

Figure S11. The average diameter of the self-assembled structures of PEG_{44}-PTA_5 measured by DLS.

Table S2. The average diameter, PDI and morphology of PEG_{44}-PTA_5 based self-assemblies.

Figure S12. SEM images of PEG_{44}-PTA_5 based self-assemblies prepared from the solvent dioxane:DMF 1:1. Yellow dash line represents the fused BCNs.

Figure S13. Average size changes of the three types of nanoparticles (PTA-Ps, PTA-LBCNs, and PTA-SBCNs) during storage at room temperature in aqueous solution.

Figure S14. Surface zeta potential of PTA-polymersomes, small and large PTA-BCNs in water.

Figure S15. Cryo-TEM (top), TEM (middle) and SEM (bottom) images of the self-assembled structures of PEG_{44}-PTA_3 prepared from the co-solvent: DMF:THF (v/v%) (a) 0:1 (b) 1:4 (c) 1:1 (d) 4:1 (e) 1:0.

Figure S16. Cryo-TEM images of PEG_{44}-PTA_3 based self-assemblies prepared from the solvent DMF:THF 1:1. Yellow dash line represents the vesicles and red dash line represent the BCNs.

Table S3. The average diameter, PDI and morphology of PEG_{44}-PTA_3 based self-assemblies.

Figure S17. The average diameter of the self-assembled structures of PEG_{44}-PTA_3 measured by DLS.

Figure S18. Morphological phase diagram of the self-assemblies of PEG_{44}-PTA_n with different hydrophobic block length and using different organic co-solvents.

Figure S19. Schematic representation of the mechanism responsible for the light capture capacity of vesicles and BCNs.

Figure S20. (a) Schematic illustration of a 2D hexagonal pore arrangement. (b) Zoomed-in cyro-TEM image taken along the pore axis direction, with the unit cell highlighted by yellow hexagons. (c) Zoomed-in cryo-TEM image showing the wavy lamellar structure, viewed along the direction perpendicular to the pore axis.

Figure S21. Absorption coefficient (808 nm) of PTA-polymersomes, small and large PTA-BCNs in water.

Figure S22. Emission spectra of PTA-polymersomes in water, large and small PTA-BCNs in water and PTA monomer in THF (Ex = 780 nm).

Figure S23. The time constant of PTA-polymersomes' heat transfer from the system, calculated with the linear time data from the cooling period.

Figure S24. The time constant of large PTA-BCNs' heat transfer from the system, calculated with the linear time data from the cooling period.

Figure S25. The time constant of small PTA-BCNs' heat transfer from the system, calculated with the linear time data from the cooling period.

Figure S26. Cryo-TEM (left) and dry-TEM (right) images of small PTA-BCNs (a) before (b) after 808 nm laser irradiation (1 W, 10 min).

Figure S27. The average diameter of small PTA-BCNs before and after 808 nm laser irradiation (1 W, 10 min) measured by DLS.

5. References

1. Materials

All reagents and solvents were purchased from commercial sources (Fluorochem, Biosolve Chimie, Fisher Scientific, Sigma-Aldrich, TCI Europe, ABCR GmbH, ThermoFisher and Rapp Polymers etc) and used without further purification. Solvents used for synthesis were purified and dried using standard techniques. Dialysis membranes were purchased from Spectra/Pro® (MWCO 12,000-14,000). Amicon Ultra-0.5 Centrifugal Filter Units (0.5 mL, 10 kDa) were from Millipore.

2. Instruments

Proton Nuclear magnetic resonance spectroscopy (NMR)

Compounds were characterized by routine proton nuclear magnetic resonance (^{1}H NMR) and fluorine nuclear magnetic resonance (^{19}F NMR) on a Bruker Avance 400 MHz Ultrashield™ spectrometer equipped with a Bruker Sample Case auto-sampler, using $CDCl_3$ as the solvent and TMS as the internal standard.

Gel permeation chromatography (GPC)

The molecular weights and dispersity of the block copolymer were characterized using a Prominence-I GPC system (Shimadzu) with a PL gel 5 μm mixed D column (Polymer Laboratories) and equipped with an RID-20A differential refractive index detector. Polystyrene standards were used for calibration. THF was used as an eluent with a flow rate of 1 mL/min.

Scanning electron microscopy (SEM)

Morphological characterization of samples was performed using SEM (FEI Quanta 200 3D FEG) at 5.00 kV voltage.

Transmission electron microscopy (TEM)

Samples were characterized by a Tecnai 20 (Thermo Fisher Scientific, type Sphera, 200 kV) which was equipped with a LaB_6 filament. To prepare the TEM samples, 5 μL sample solution (in Milli-Q water) was pipetted on the TEM carbon-coated copper grids. After drying at ambient conditions, TEM images were captured using a 4k × 4k Ceta CCD camera.

Cryogenic transmission electron microscopy (Cryo-TEM)

Cryo-TEM was conducted on the TU/e CryoTitan equipped with a field-emission gun operating at 300 kV, an autoloader station and a post-column Gatan. To prepare the cryo-TEM samples, 3 μL sample solution was pipetted on the grid (Lacey carbon coated, R2/2, Cu, EM sciences) and blotted in a Vitrobot MARK IV at 100% humidity to remove the excess solution. (The pore sizes of small and large PTA-BCNs were determined from Cryo-TEM images by analysis of 30 different assemblies)

Small angle X-ray scattering (SAXS)

Small angle X-ray scattering measurements were performed on a SAXSLAB Ganesha system. The Ganesha system consisted of a high brilliance copper Genix3D Microfocus Source, wavelength 1.5418Å, a motorized collimation system and a Pilatus 300K solid-state 2D-detector. The whole system was at vacuum, pressure of about 0.2 mbar. The sample-to-detector distance was varied between 80 and 1450mm resulting in a q-range between 2.5 and 0.003$Å^{-1}$. A silver behenate standard was used to verify the sample to detector distance. The samples were prepared in 2mm quartz glass capillaries. Measurements were obtained in transmission mode with an exposure time between 4-6 hours. The SAXS data was collected and analyzed with SAXSGUI and SAXSutilities software. The background scatter of the solvent buffer (Milli-Q water) was subtracted resulting in the SAXS data shown. Finally, the SAXS data was analyzed to obtain the final scattering curve.

Dynamic light scattering measurements (DLS)

The hydrodynamic size of the samples was characterized using a Zetasizer (model Nano ZSP, Malvern Instruments) equipped with a 633 nm He-Ne laser and an avalanche photodiode detector. To process and analyze the data, Zetasizer software was used.

UV-vis-NIR spectrophotometer

The absorbance of sample solutions was characterized using a Cary 3500 UV-vis-NIR spectrophotometer (Agilent).

Thermometer

Real time temperatures of sample solutions upon laser irradiation were measured using a thermometer (CHAUVIN ARNOUX C.A 1823).

Infrared camera

Thermal imaging pictures of sample solutions were taken with a camera (FLIR E54).

3. Methods

3.1 Synthesis of photothermal block copolymer (PEG_{44}-PTA_3 and PEG_{44}-PTA_5).

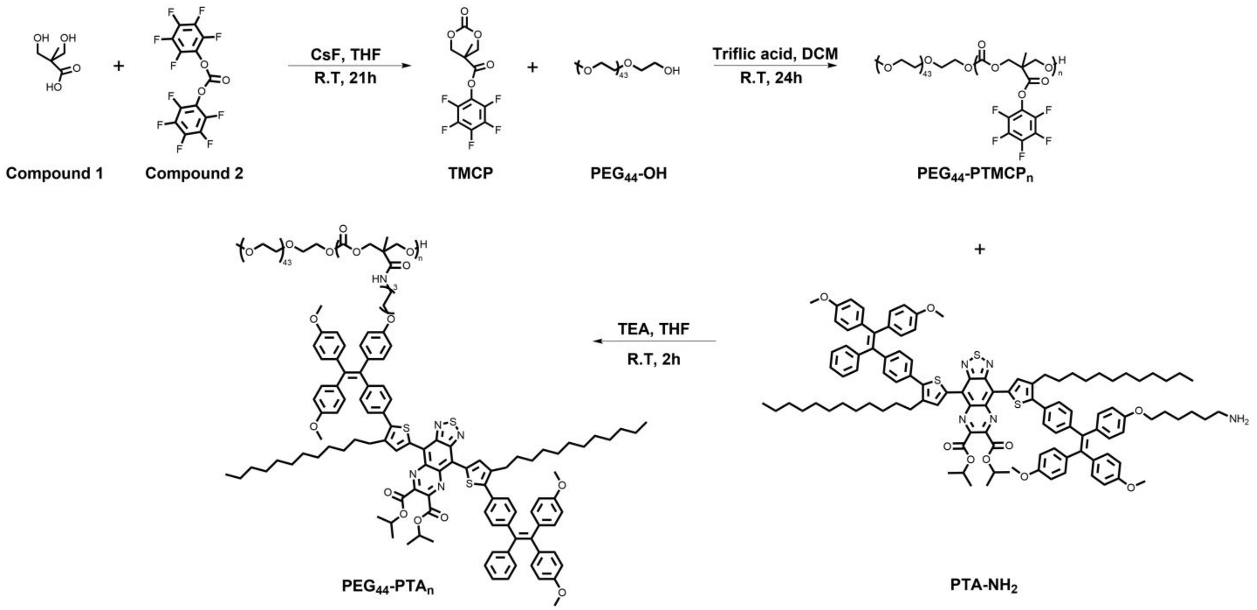

Scheme S1. Synthesis route of photothermal block copolymers (PEG_{44}-PTA_3 and PEG_{44}-PTA_5).

TMCP: A 250 mL round bottom flask was charged with 2,2-bis(hydroxymethyl)propionic acid (3.00 g, 22 mmol), bis(pentafluorophenyl) carbonate (21.70 g, 55 mmol), CsF (0.70 g, 4.6 mmol), and 120 mL of anhydrous THF. The reaction mixture was stirred at room temperature for 24 h. After completion, the solvent was removed under reduced pressure. The resulting residue was dissolved in methylene chloride and stirred for 10 min, during which a byproduct precipitated and was removed by filtration. The filtrate was washed with aqueous sodium bicarbonate and water, then dried over anhydrous $MgSO_4$. After filtration, the solvent was removed under vacuum, and the crude product was recrystallized from an ethyl acetate/hexane mixture to afford TMC-OPh_5 (TMCP) as a white crystalline powder. Yield: 4.41 g (60 % yield). ^1H NMR (400 MHz, $CDCl_3$), δ (ppm) = 4.86-4.84 (d, J = 10.8 Hz, 2H,), 4.39-4.36 (d, J = 10.8 Hz, 2H,), 1.56 (s, 3H); ^{19}F NMR (376 MHz, $CDCl_3$), δ (ppm) = 152.79 (m, 2F), 156.11 (t, 1F), 161.18 (m, 2F).[1]

PEG_{44}-$PTMCP_3$: Monomethyl-PEG-OH macro-initiator 2 kDa (984 mg, 0.5 mmol) and dry TMCP (652 mg, 2 mmol) were added into a round bottom flask. Under argon flow, 3 mL dry DCM (1 M with respect to TMCP) was added into the flask. The TMCP only partially dissolves at this concentration. Triflic acid (70 μL, 1 mmol) was added to the stirring solution. As the reaction proceeded, the undissolved TMCP slowly went into solution. The reaction was monitored by ^1H NMR spectroscopy. Once the reaction was complete, the polymer was precipitated into hexanes. The crude polymer was then re-dissolved in minimal amount of DCM and precipitated into diethyl ether, isolated, and freeze dried to obtain the product, and kept in the freezer until being used. ^1H NMR (400 MHz, $CDCl_3$): δ (ppm) = 4.48-4.44 (m, 12H), 3.61-3.66 (m, 176H), 3.38 (s, 3H), 1.49 (s, 9H); ^{19}F NMR (376 MHz, $CDCl_3$), δ (ppm) = 153.04 (m, 2F), 157.05 (t, 1F), 161.99 (m, 2F).[2]

PEG_{44}-$PTMCP_5$: PEG_{44}-$PTMCP_5$ was synthesized *via* a similar procedure as PEG_{44}-$PTMCP_3$. ^{1}H NMR (400 MHz, $CDCl_3$): δ (ppm) = 4.48-4.44 (m, 20H), 3.61-3.66 (m, 176H), 3.38 (s, 3H), 1.49 (s, 15H); ^{19}F NMR (376 MHz, $CDCl_3$), δ (ppm) = 153.00 (m, 2F), 156.99 (t, 1F), 161.97 (m, 2F).

PEG_{44}-PTA_3: PEG_{44}-$PTMCP_3$ (59 mg, 0.02 mmol) was dissolved in 1 mL dry THF and cooled on an ice bath. Next, a 0.5 mL THF solution containing PTA-NH_2 (121 mg, 0.07 mmol, 1.15 equiv. with respect to the pentafluorophenyl ester) and triethylamine (TEA, 14 μL, 0.05 mmol, 1.15 equiv. with respect to the pentafluorophenyl ester) were dropwise added. The ice bath was removed and the mixture was allowed to stir for an additional 90 min. The reaction was monitored by ^{1}HNMR and ^{19}F NMR spectroscopy. After the complete conversion of the pentafluorophenyl ester, the reaction solution was precipitated into diethyl ether three times. Then the green solid was isolated and freeze dried to obtain PEG_{44}-PTA_3 (72 mg, yield: 47%). ^{1}H NMR (400 MHz, $CDCl_3$): δ (ppm) = 8.97 (s, 6H), 7.32-7.34 (m, 12H), 7.08-7.15 (m, 27H), 6.95-6.99 (m, 30H), 6.64-6.68 (m, 30H), 4.29 (s, 12H), 3.86-3.92 (m, 6H), 3.71-3.77 (m, 36H), the protons of PEG 3.61-3.66 (m, 176H), terminal methyl unit 3.38 (s, 3H), 2.74-2.79 (m, 12H), 1.43-1.45 (d, J = 8 Hz, 36H), 1.21-1.32 (m, 111H), 0.86-0.88 (m, 18H).

PEG_{44}-PTA_5: PEG_{44}-PTA_5 was synthesized *via* a similar procedure as PEG_{44}-PTA_3. ^{1}H NMR (400 MHz, $CDCl_3$): δ (ppm) = 8.97 (s, 10H), 7.32-7.34 (m, 20H), 7.08-7.15 (m, 45H), 6.95-6.99 (m, 50H), 6.64-6.68 (m, 50H), 5.39-5.45 (m, 10H), 4.29 (s, 20H), 3.86-3.92 (m, 10H), 3.71-3.77 (m, 60H), the protons of PEG 3.61-3.66 (m, 176H), terminal methyl unit 3.38 (s, 3H), 2.74-2.79 (m, 20H), 1.43-1.45 (d, J = 8 Hz, 60H), 1.21-1.32 (m, 185H), 0.86-0.88 (m, 30H).

3.2 Preparation of self-assemblies *via* solvent switch method.

1mg photothermal block copolymer was dissolved in 0.5 mL organic solvent in a glass vial with a magnetic stirring bar. After stirring for 10 min, 0.5 mL of ultrapure Milli-Q water was added *via* a syringe pump (Chemyx, Inc., Fusion 100, KR Analytical Limited, Stafford, TX, USA) with a speed of 0.25 mL/h. The resulting cloudy solution was transferred into a 2 mL/cm pre-hydrated dialysis bag (MWCO 12,000-14,000, Spectra/Pro®, Rancho Dominguez, CA, USA) for dialysis against Milli-Q water at 4 °C with a water change after 1 h, followed by dialysis for at least 24 h.[2]

3.3 Photothermal performance of PTA-polymersomes, large PTA-BCNs and small PTA-BCNs.

Photothermal performance: Samples were continuously irradiated with an 808 nm NIR laser for 10 min. The temperature was measured every second using a digital thermometer with a thermocouple probe. The IR thermal images of the sample tubes were also recorded.[3]

Photothermal stability: For morphology stability studies, a solution of small PTA-BCNs (1mg/mL) was irradiated with an 808 nm laser for 10 min, and the structures were analyzed with Cryo-TEM. For photothermal stability studies, the temperatures of the sample solutions were recorded during five circles of heating and cooling. In each heating-cooling circle, the NIR laser was first used to irradiate the samples for 10 min, then the laser was turned off, and the samples were naturally cooled down for 10 min.

3.4 Calculation of photothermal conversion efficiency

The photothermal conversion efficiency (η) of the PTA-nanoparticles was calculated according to the reported method.[3] The detailed calculation was carried out using the following equations:

$$\eta = \frac{hA(T_{max} - T_{surr}) - Q_{dis}}{I(1 - 10^{-A_{808}})} \quad \dots (1)$$

$$hA = \frac{mC_{water}}{\tau_s} \quad \dots (2)$$

$$t = -\tau_s ln\theta = -\tau_s \ln\left(\frac{T - T_{surr}}{T_{max} - T_{surr}}\right)$$

... (3)

$$Q_{dis} = \frac{mC_{water}(T_{\max\,(water)} - T_{surr})}{\tau_{water}}$$

... (4)

where h is the heat transfer coefficient, A is the surface area of the container, m is the mass of the solution, Q_{dis} is heat dissipation loss, τ_s is a system time constant, C_{water} is specific heat capacity of water, I is incident laser power, η is the photothermal conversion efficiency, A_{808} indicates the absorbance of solution, T_{surr} is the surrounding temperature, T_{max} and $T_{max(water)}$, are the max temperature of solution and water, respectively.

4. Supplementary figures

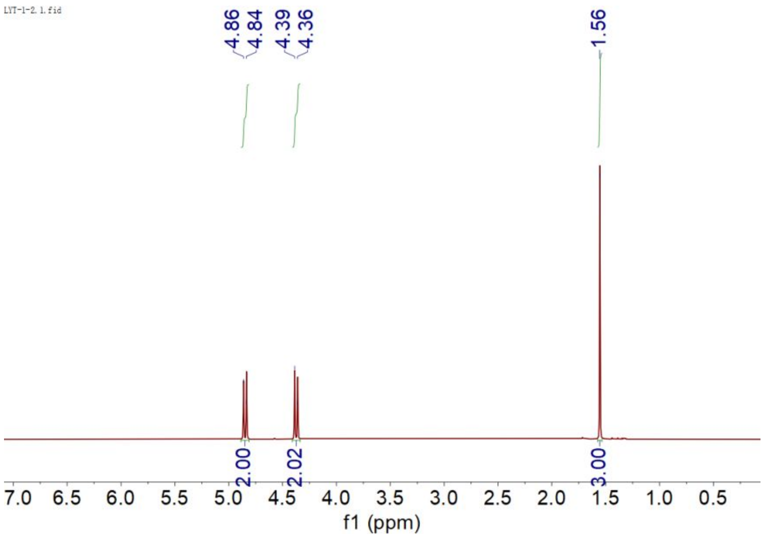

Figure S1. Representative ^{1}H NMR spectrum of TMCP (400 MHz, 298 K, $CDCl_3$).

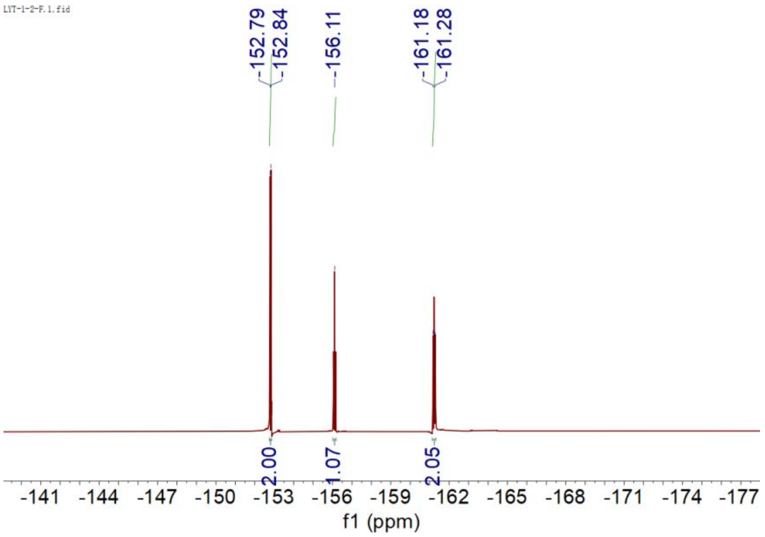

Figure S2. Representative ^{19}F NMR spectrum of TMCP (376 MHz, 298 K, $CDCl_3$).

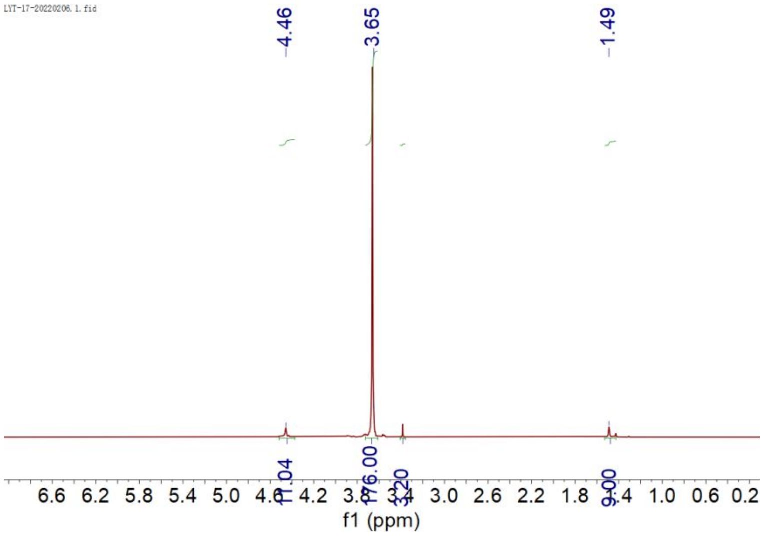

Figure S3. Representative ^{1}H NMR spectrum of PEG_{44}-$TMCP_3$ (400 MHz, 298 K, $CDCl_3$).

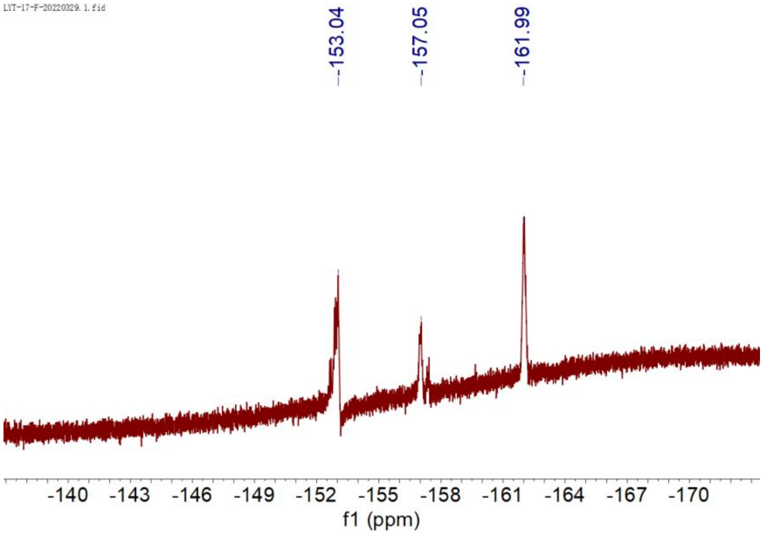

Figure S4. Representative ^{19}F NMR spectrum of PEG_{44}-$TMCP_3$ (376 MHz, 298 K, $CDCl_3$).

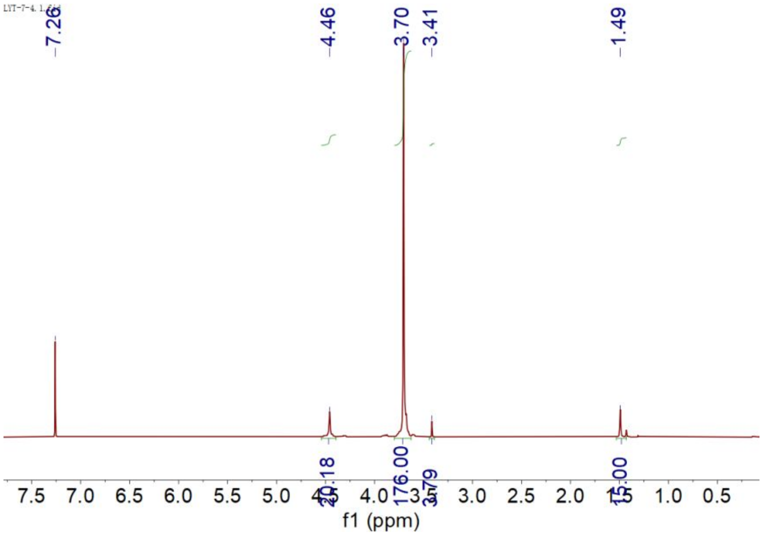

Figure S5. Representative ^{1}H NMR spectrum of PEG_{44}-$TMCP_5$ (400 MHz, 298 K, $CDCl_3$).

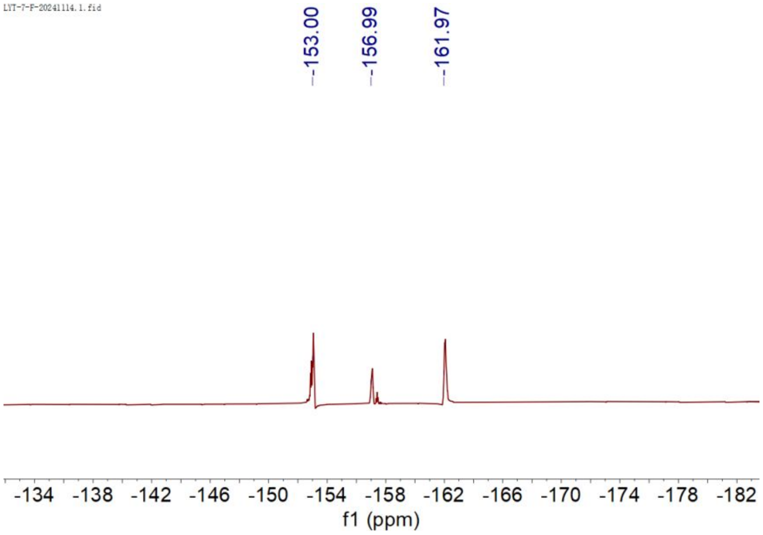

Figure S6. Representative ^{19}F NMR spectrum of PEG_{44}-$TMCP_5$ (376 MHz, 298 K, $CDCl_3$).

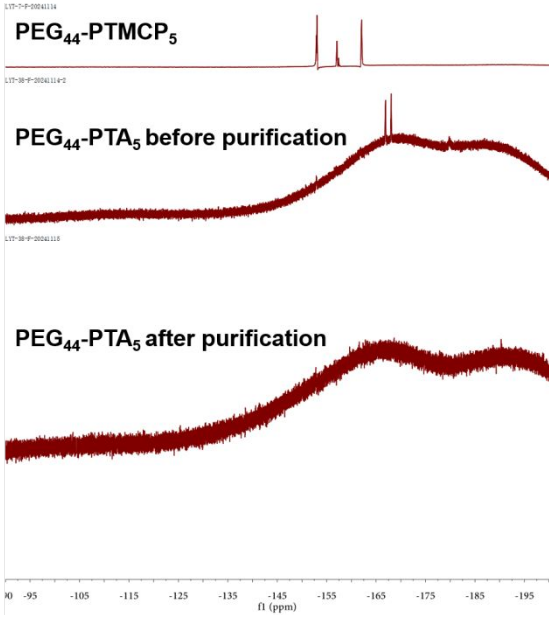

Figure S7. Representative ^{19}F NMR spectrum of PEG_{44}-$PTMCP_5$ (top), PEG_{44}-PTA_5 before purification (middle) and PEG_{44}-PTA_5 after purification (down).

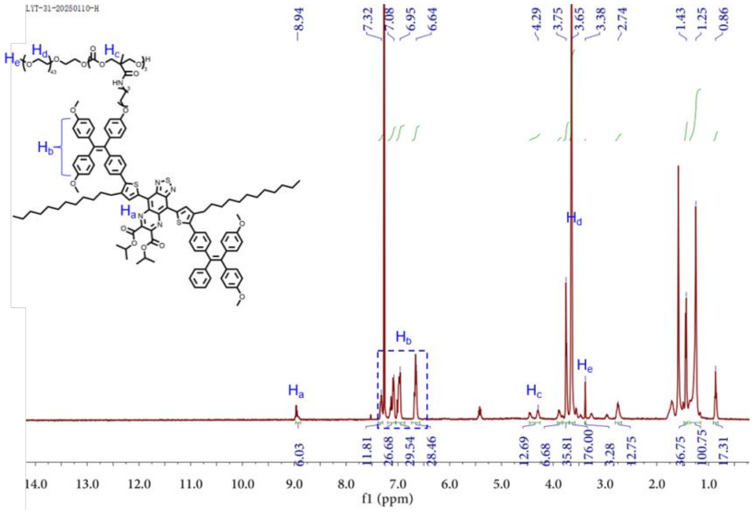

Figure S8. ^{1}H NMR spectrum of PEG_{44}-PTA_3 (400 MHz, 298 K, $CDCl_3$).

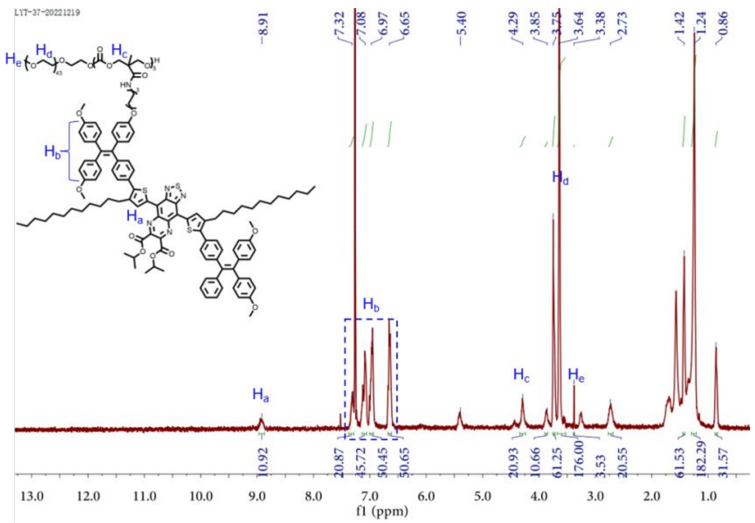

Figure S9. ^{1}H NMR spectrum of PEG_{44}-PTA_5 (400 MHz, 298 K, $CDCl_3$).

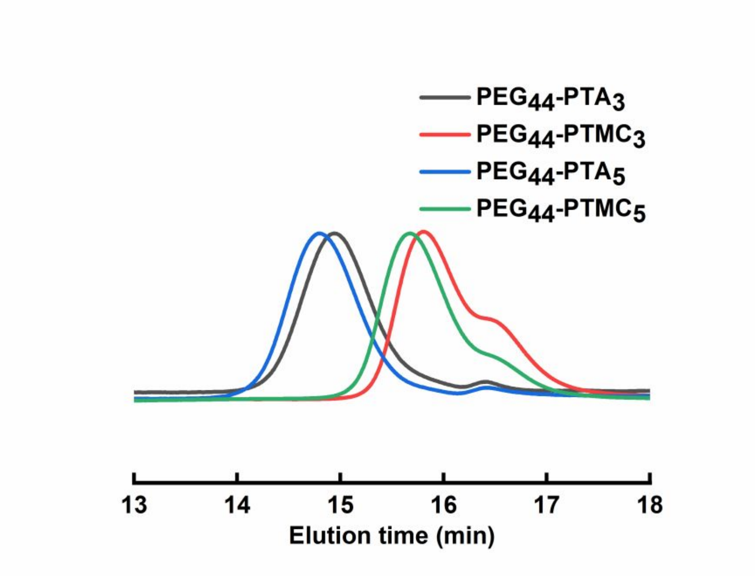

Figure S10. GPC trace of PEG_{44}-$PTMCP_3$, PEG_{44}-$PTMCP_5$, PEG_{44}-PTA_3 and PEG_{44}-PTA_5.

Table S1. GPC analysis of block copolymers.

Composition	Mn / kDa	Mw / kDa	PDI (Đ)
**PEG_{44}-$PTMCP_3$**	2497	3054	1.22
**PEG_{44}-$PTMCP_5$**	3134	3743	1.19
**PEG_{44}-PTA_3**	7327	8078	1.10
**PEG_{44}-PTA_5**	8884	10011	1.12

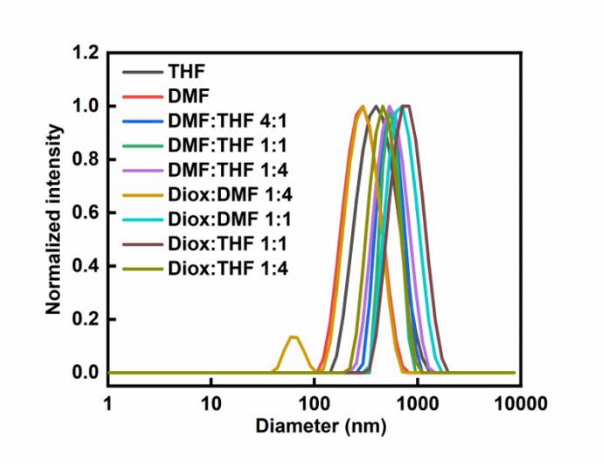

Figure S11. The average diameter of the self-assembled structures of PEG_{44}-PTA_5 measured by DLS.

Table S2. The average diameter, PDI and morphology of PEG_{44}-PTA_5 based self-assemblies.

Ratio of solvent (v/v%)	Average diameter (nm)	PDI	Morphology
**Pure THF**	384 ± 3	0.13 ±0.02	Vesicles
**DMF:THF 1:4**	560 ± 10	0.15 ±0.05	Vesicles
**DMF:THF 1:1**	550 ± 5	0.06 ±0.02	BCNs
**DMF:THF 4:1**	535 ± 1	0.05± 0.03	BCNs
**Pure DMF**	263 ± 4	0.09 ± 0.04	BCNs
**DMF:Diox 4:1**	235 ± 2	0.31± 0.10	BCNs
**DMF:Diox 1:1**	737 ± 14	0.07 ± 0.02	BCNs
**THF:Diox 1:1**	784 ± 12	0.17± 0.02	Vesicles
**THF:Diox 4:1**	426 ± 3	0.19 ± 0.02	Vesicles

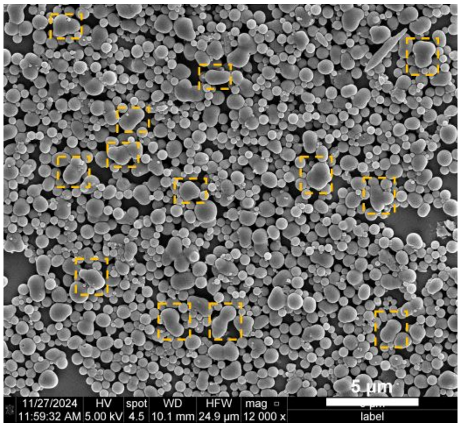

Figure S12. SEM image of PEG_{44}-PTA_5 based self-assemblies prepared from the solvent dioxane:DMF 1:1. Yellow dashed boxes indicate the fused BCNs.

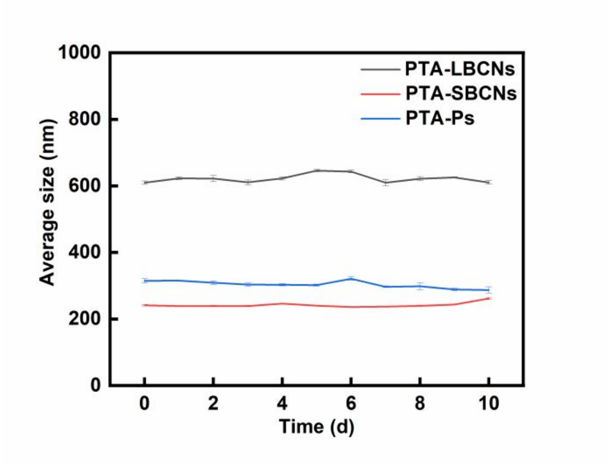

Figure S13. Average size changes of the three types of nanoparticles (PTA-Ps, PTA-LBCNs, and PTA-SBCNs) during storage at room temperature in aqueous solution.

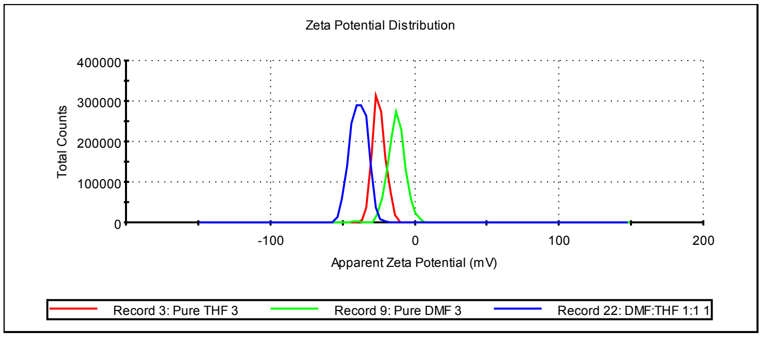

Figure S14. Surface zeta potential of PTA-polymersomes, small and large PTA-BCNs in water.

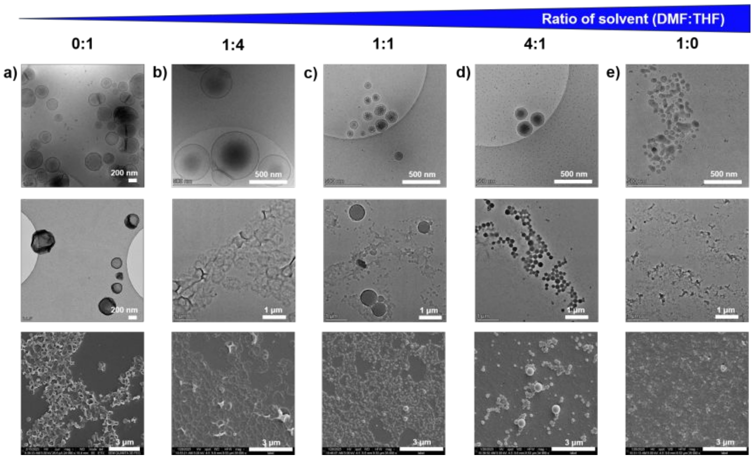

Figure S15. Cryo-TEM (top), TEM (middle) and SEM (bottom) images of the self-assembled structures of PEG_{44}-PTA_3 prepared from the co-solvent: DMF:THF (v/v%) (a) 0:1 (b) 1:4 (c) 1:1 (d) 4:1 (e) 1:0.

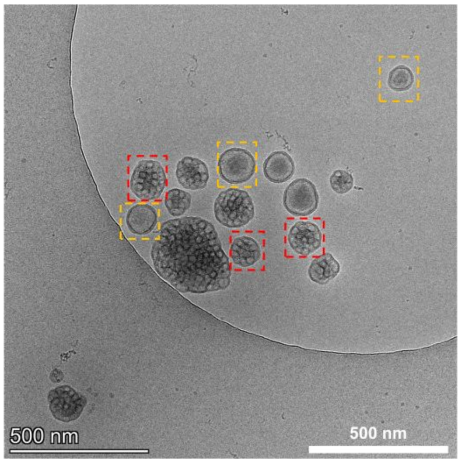

Figure S16. Cryo-TEM images of PEG_{44}-PTA_3 based self-assemblies prepared from the solvent DMF:THF 1:1. Yellow dashed boxes indicate the vesicles and the red ones the BCNs.

Table S3. The average diameter, PDI and morphology of PEG_{44}-PTA_3 based self-assemblies.

Ratio of solvent (v/v%)	Average diameter (nm)	PDI	Morphology
**Pure THF**	417 ± 1	0.07± 0.03	Vesicles
**DMF:THF 1:4**	1122 ± 23	0.20 ± 0.01	Vesicles
**DMF:THF 1:1**	182 ± 2	0.29 ± 0.03	Vesicles and BCNs
**DMF:THF 4:1**	221± 1	0.18 ± 0.01	Micelles and BCNs
**Pure DMF**	138 ± 1	0.20 ± 0.01	Fusion BCNs

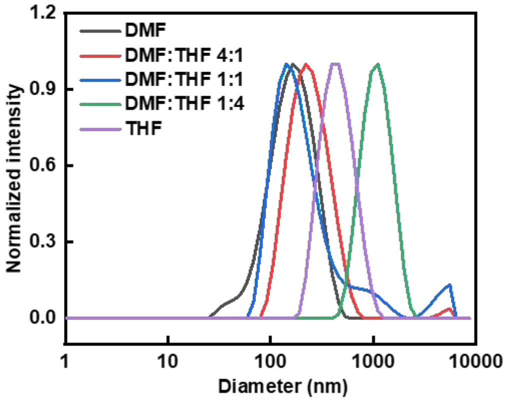

Figure S17. The average diameter of the self-assembled structures of PEG_{44}-PTA_3 prepared from different solvents, measured by DLS.

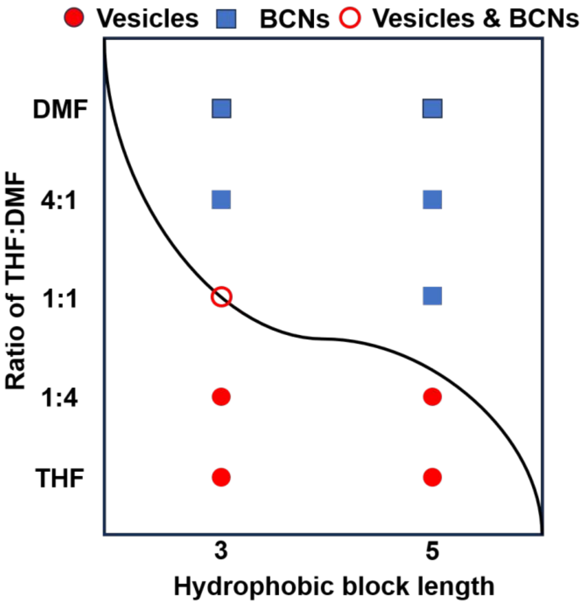

Figure S18. Morphological phase diagram of the self-assemblies of PEG_{44}-PTA_n with different hydrophobic block length and using different organic co-solvents.

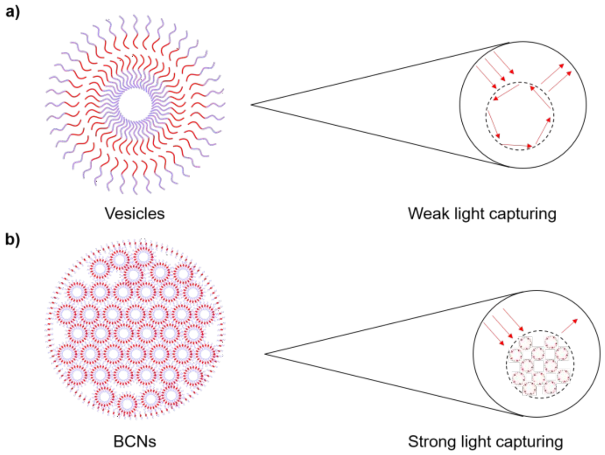

Figure S19. Schematic representation of the mechanism responsible for the light capture capacity of vesicles and BCNs

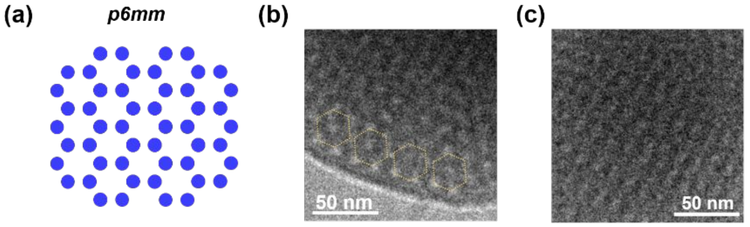

Figure S20. (a) Schematic illustration of a 2D hexagonal pore arrangement. (b) Zoomed-in cyro-TEM image taken along the pore axis direction, with the unit cell highlighted by yellow hexagons. (c) Zoomed-in cryo-TEM image showing the wavy lamellar structure, viewed along the direction perpendicular to the pore axis.

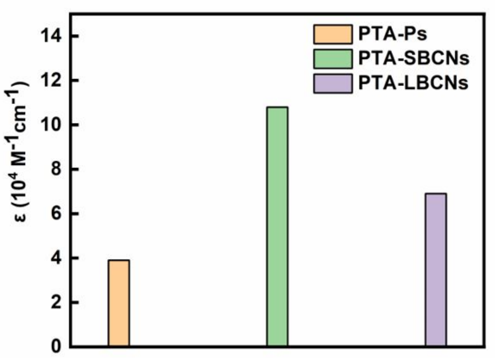

Figure S21. Absorption coefficient (808 nm) of PTA-polymersomes, small and large PTA-BCNs in water.

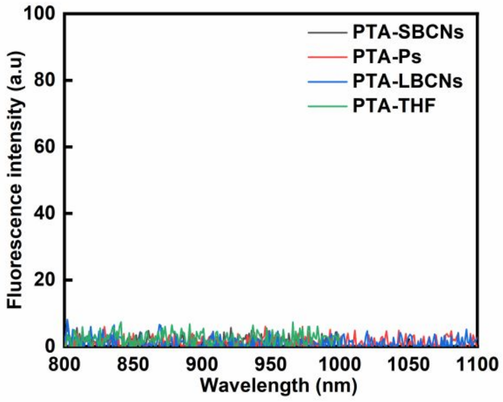

Figure S22. Emission spectra of PTA-polymersomes in water, large and small PTA-BCNs in water and PTA monomer in THF (Ex = 780 nm).

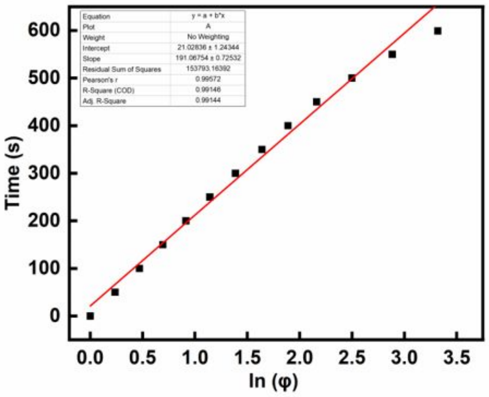

Figure S23. The time constant of PTA-polymersomes' heat transfer from the system, calculated with the linear time data from the cooling period.

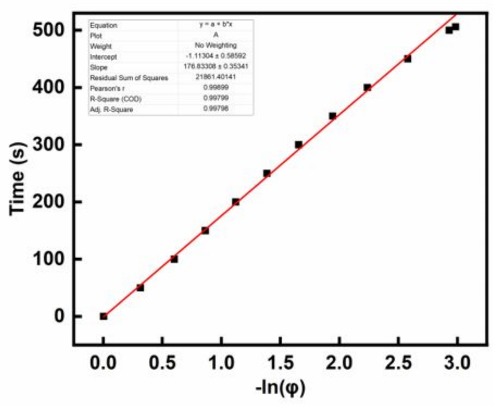

Figure S24. The time constant of large PTA-BCNs' heat transfer from the system, calculated with the linear time data from the cooling period.

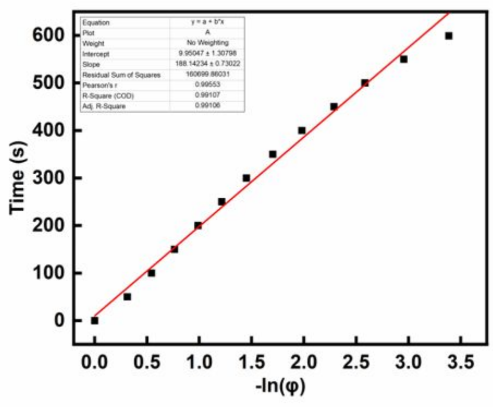

Figure S25. The time constant of small PTA-BCNs' heat transfer from the system, calculated with the linear time data from the cooling period.

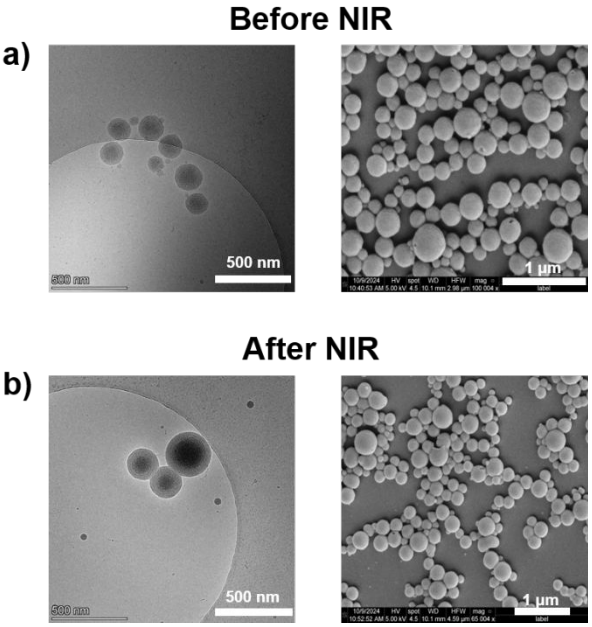

Figure S26. Cryo-TEM (left) and dry-TEM (right) images of small PTA-BCNs (a) before (b) after 808 nm laser irradiation (1 W, 10 min).

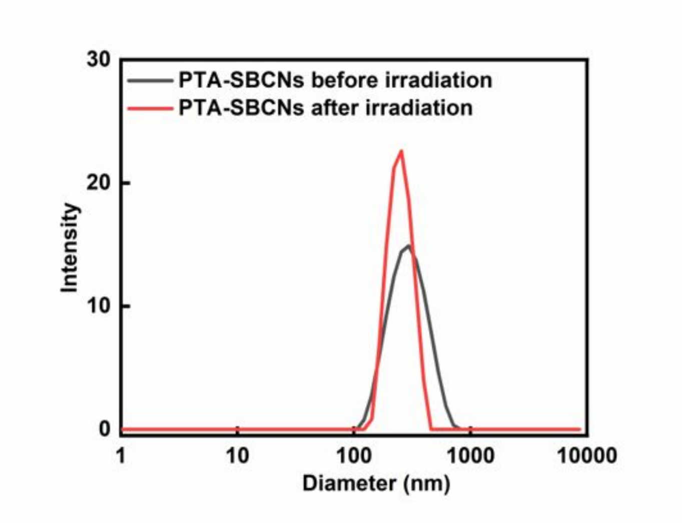

Figure S27. The average diameter of small PTA-BCNs before and after 808 nm laser irradiation (1 W, 10 min) measured by DLS.

5. References

1. Cao, S.; Shao, J.; Wu, H.; Song, S.; De Martino, M. T.; Pijpers, I. A. B.; Friedrich, H.; Abdelmohsen, L. K. E. A.; Williams, D. S.; Van Hest, J. C. M. Photoactivated nanomotors via aggregation induced emission for enhanced phototherapy. *Nat. Commun*. **2021**, *12*, 1-10.
2. Luo, Y.; Wu, H.; Zhou, X.; Wang, J.; Er, S.; Li, Y.; Welzen, P.; Oerlemans, R. A. J. F.; Abdelmohsen, L. K. E. A.; Shao, J and van Hest, J. C. M. Polymer vesicles with integrated photothermal responsiveness. *J. Am. Chem. Soc.* **2023**, *145*, 20073-20080.
3. Liu, S.; Zhou, X.; Zhang, H.; Ou, H.; Lam, J. Y.; Liu, Y.; Shi, L.; Ding, D.; Tang, B. Z. Molecular Motion in Aggregates: Manipulating TICT for Boosting Photothermal Theranostics. *J. Am. Chem. Soc*. **2019**, *141*, 5359-5368.
